# Atrial Fibrillation After Elective Isolated Coronary Artery Bypass Graft Surgery

**DOI:** 10.21470/1678-9741-2023-0337

**Published:** 2024-05-10

**Authors:** Mesut Engin, Ufuk Aydın, Yusuf Ata

**Affiliations:** 1 Department of Cardiovascular Surgery, University of Health Sciences, Bursa Yuksek Ihtisas Training and Research Hospital, Yildirim, Bursa, Turkey. E-mail: mesut_kvc_cor@hotmail.com; 1 Department of Cardiovascular Surgery, University of Health Sciences, Bursa Yuksek Ihtisas Training and Research Hospital, Yildirim, Bursa, Turkey; 1 Department of Cardiovascular Surgery, University of Health Sciences, Bursa Yuksek Ihtisas Training and Research Hospital, Yildirim, Bursa, Turkey

Dear Editor,

We have read the article by Apaydin et al.^[1]^ entitled “Could We Predict POAF With a Simple Ambulatory Oscillometry Evaluating Aortic Stiffness?” with great interest. First of all, we congratulate the authors for their valuable contribution to the literature. However, we would like to discuss some points about postoperative atrial fibrillation (PoAF) and its risk factors.

In that prospective study, the authors investigated the effect of aortic stiffness (AS) status on PoAF in patients who underwent isolated coronary artery bypass grafting^[1]^. AS is an important sign of aging and atherosclerosis. It is known that this condition is correlated with hypertension (HT), end-stage renal disease, diabetes mellitus, and being over 70 years of age^[2]^. In the study, pulse wave velocity (PWV) value was found to be significantly correlated with the development of PoAF (odds ratio: 1.561; 95% confidence interval: 1.119-2.177). However, the frequency of HT was found to be quite similar in patients with and without PoAF (47.4% *vs.* 50%). While PoAF and PWV were significantly correlated in these groups, why do the authors think the frequency of HT was similar between the groups? HT is an important risk factor for PoAF and has taken its place in important atrial fibrillation risk assessment scores^[3,4]^. In addition, data on the prevalence of coronary artery disease in the study were given as “graft count”. The SYNTAX score I is an important indicator of the severity of coronary artery disease and its relationship with PoAF is known^[5]^. Were the SYNTAX score I values similar in that patient group?

PoAF is affected by many other factors perioperatively. These include the use of blood products, positive inotropic agents, and especially early medical treatments^[6,7]^. How was the postoperative medical treatment of the patients in the study group performed? Did all patients start on early metoplorol and statin therapy? We believe that clarification of these points will increase the value of the study.

Finally, we would like to emphasize the importance of electrocardiography (ECG) follow-up in the diagnosis of PoAF in the postoperative period. The most important problem in retrospective PoAF studies is that the diagnosis was missed in some patients who did not show clinical complaints and developed PoAF^[5,8]^. However, the study was planned prospectively. What kind of problems precluded the authors from performing postoperative ECG monitoring with continuous telemetry in the clinic? We think that this is an important limiting point. Perhaps this problem will be overcome in the future with the developing technology and the use of smartwatches becoming widespread in society^[9]^.
